# Corrosion Development of Carbon Steel Grids and Shear Connectors in Cracked Composite Beams Exposed to Wet–Dry Cycles in Chloride Environment

**DOI:** 10.3390/ma11040479

**Published:** 2018-03-22

**Authors:** Wen Xue, Ju Chen, Fei Xu, Ao-yu Jiang

**Affiliations:** 1 School of Civil Engineering and Architecture, Zhejiang University of Science and Technology, Hangzhou 310023, China; xuewen@zust.edu.cn; 2 Department of Civil Engineering, Zhejiang University, Hangzhou 310058, China; cecj@zju.edu.cn (J.C.); ijstructe@zju.edu.cn (A.J.); 3 Department of Civil and Environmental Engineering, Hong Kong Polytechnic University, Hong Kong, China

**Keywords:** corrosion, chloride, crack, pitting, cross-section loss, reinforcement, shear connectors

## Abstract

The corrosion development of the reinforcement and shear stud connectors in the cracked steel–concrete composite beams under the salt-fog wet–dry cycles is presented in this investigation. Seven identical composite beams with load-induced concrete cracks were exposed to an aggressive chloride environment. The reinforcement and shear connectors were retrieved after specimens underwent a specified number of wet–dry cycles to obtain the corrosion pattern and the cross-section loss at different exposure times and their evolutions. The crack map, the corrosion pattern and the cross-section loss were measured and presented. Based on the experimental results, the influence of crack characteristics, including crack widths, orientations and positions on the corrosion rate and distribution, were accessed. Moreover, the effects of the connecting weldments on the corrosion initiations and patterns were analyzed. It was shown that the corrosion rate would increase with the number of wet–dry cycles. The characteristics of load-induced cracks could have different influences on the steel grids and shear stud connectors. The corrosion tended to initiate from the connecting weldments, due to the potential difference with the parent steel and the aggressive exposure environment, leading to a preferential weldment attack.

## 1. Introduction

**Figure 1:**
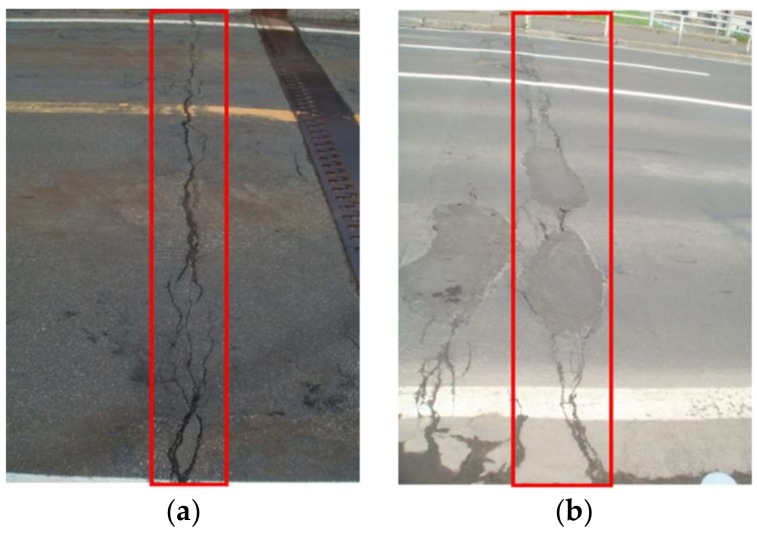
Cracks in the hogging moment region of steel-composite bridges (**a**: in Hokkaido, Japan; **b**: in Sapporo, Japan).

Steel–concrete composite beams process the superior mechanical performance on the load-carrying capacity and the flexural stiffness by utilizing advantages of both steel and concrete materials from composite actions by the shear connectors. Therefore, they have been extensively applied to buildings and bridges. However, the concrete slab in the hogging moment region will be subjected to a tensile-dominant stress state leading to unfavorable flexural cracks. Figure 1 presents the typical cracks in the hogging moment region of the composite bridges. These load-induced cracks on the concrete slab surfaces bring aggressive agents into the reinforcement and shear connectors, leading to a series of durability issues, especially when structures are exposed to a chloride environment.

It has been well established that steel reinforcement corrosion in concrete structures will affect both serviceability and safety [1,2,3], due to corrosion-induced cracks, concrete cover spalling, cross-section loss and degradation of steel–concrete bond strength [4]. Considerable investigations have been conducted to evaluate the influence of different factors, such as load-induced cracks and exposure conditions, on the development of chloride-induced corrosion on the rebars in reinforced concrete (RC) structures [5,6,7,8,9]. The initial corrosion can be accelerated by the existing cracks which provide the access for detrimental substances, such as oxygen, chlorides and water, to the rebar surfaces [10,11,12]. However, little research has focused on the corrosion evolution of cracked composite beams. The weld connecting methodology used for connecting the reinforcement in two directions and the shear connectors with steel beams is quite different from RC structure cases. Preferential weldment corrosion has been observed and extensively studied in the petroleum and petrochemical industry, chemical processing industry, marine shipping industry, and so on [13], due to the inherent property of weld metals, the complicated microstructure of weldment and the thermal history effects during the welding process. This can therefore lead to a distinct corrosion pattern and evolution for composite beams. Moreover, the experimental investigations on the pull-out [14,15] and fatigue [16] behaviour of corroded stud shear connections indicated that the corrosion rate of shear studs had significant effects on the ultimate shear resistance and fatigue life. Essentially, the assessment of the corrosion patterns and evolution will also provide a solid basis for understanding and quantifying the mechanical performance of corroded composite beams.

In this study, seven identical steel–concrete composite beams were fabricated, pre-loaded to generate load-induced cracks and exposed to different numbers of wet–dry cycles within a chloride environment. The crack maps, corrosion patterns and cross-section losses of the reinforcement and shear connectors at different exposure times were retrieved and compared. The influence of the load-induced crack characteristics, including the crack width, orientation and position, on the corrosion pattern and the cross-section loss at different exposure times, was evaluated. Moreover, the weldment effects on the corrosion development of the reinforcement and studs were demonstrated and analyzed. Based on this study, new knowledge on the corrosion development of composite beams will be presented, which will promote the application of composite structures in aggressive chloride environments.

## 2. Experimental Section

### 2.1. Test Specimens 

**Figure 2:**
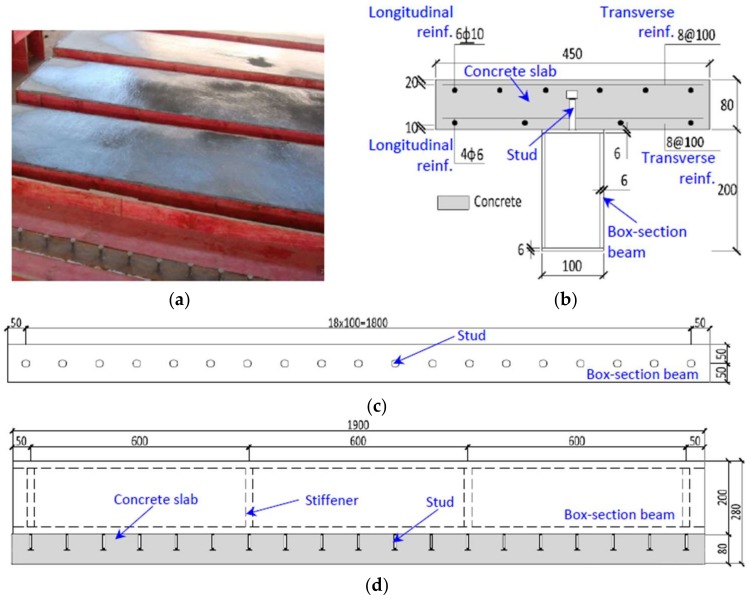
Details of the composite test specimens. (**a**) Casting concrete; (**b**) transverse view; Top view; (**d**) Front view.

In total, seven identical steel–concrete composite beams were constructed to facilitate the investigation on the corrosion evolution of stud shear connectors under indoor-accelerate environmental exposure. The design details of the composite beams are shown in Figure 2. The length, width and depth of each composite beam was kept constant at 1900 mm, 450 mm (measured from a concrete slab) and 280 mm, respectively. There were four steel stiffeners arranged inside the plate-welded-box girders to avoid the local buckling of the tube-wall. A single line of studs [17] was welded on the top face of the box girder, with each stud spaced at a constant distance of 100 mm. The nominal diameter and height of the stud shank were 10 mm and 50 mm, respectively. In the concrete slab, two reinforcement grids were arranged at both the top and bottom with a concrete cover of 20 mm and 10 mm, respectively. Six longitudinal hot-rolled ribbed bars (HRBs) with a nominal diameter of 10 mm were arranged at the top of the concrete slab to resist the hogging moment, whilst four hot-rolled plain bars (HPBs) with a nominal diameter of 4 mm were at the bottom. For the transverse direction, the HPBs with a nominal diameter of 8 mm were placed with a constant spacing of 100 mm. The specimens were labeled according to the exposure periods shown in Table 1. The letters CB, a number, the letter M and the letter R referred to the composite beam, exposure time, month and repeating specimens, respectively. For example, CB12MR stood for the repeat specimen of the composite beam with a total exposure time of twelve months.

### 2.2. Material Properties and Measurements

The composite beams were casted and cured in the position shown in Figure 2a. The concrete composition is summarized in Table 2. The concrete consisted of ordinary Portland cement, water, sand and stone, with maximum nominal size of 14 mm. The concrete mixture with a water/cement ratio of 0.4 resulted in a slump value of 60 mm. The 28-day strength of the concrete tested from 150-mm-cubic specimens was 49.6 MPa [18].

The mechanical and chemical properties of the stud, reinforcement and box girder used in the current investigation follow the Chinese code for the design of concrete structures (GB 50010-2010) [19] and the Chinese code for the design of steel structures (GB50017-2003) [20]. The mechanical properties are summarized in Table 3. The yield strengths for the studs and rebars were derived from 0.2% proof stress since there was no obvious yield plateau in their testing strain-stress curves. The box girders were factory manufactured according to GB50017-2003 [20]. The studs were also welded in the factory using a drawn arc stud welding machine (model number RSN-1600, made in China). The welding parameters for a 10-mm stud are as follows: welding current: 550 A and a time of 0.35 s; these parameters were determined according to the stud diameter. Welding trials were conducted to secure the welding quality for both studs and welded box-beams and manifested the satisfactory weld quality and the controllability of the welding processes.

### 2.3. Pre-Cracked Test and Exposure Conditions

**Figure 3:**
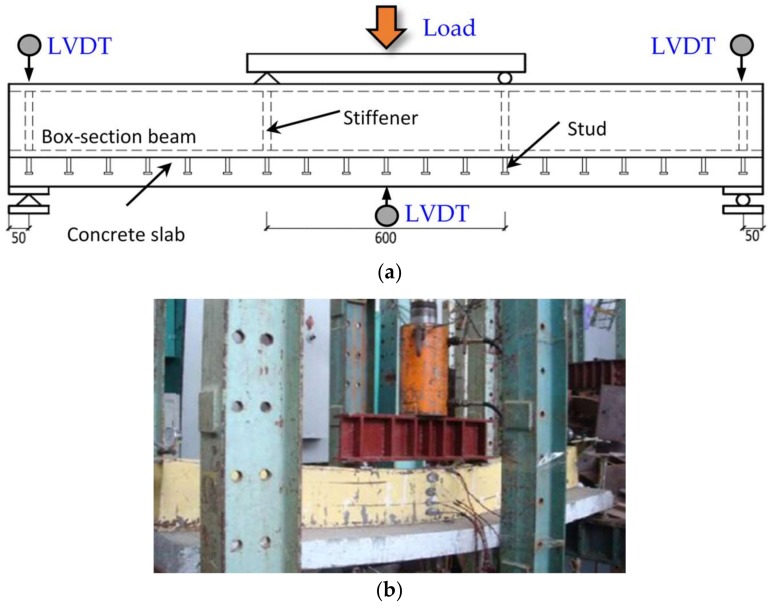
Test set-up for creating pre-cracks under the negative moment. (**a**) Schematic diagram; (**b**) test photo.

After 28-day curing in the laboratory environment, all the composite beams were loaded under a negative moment of 70% design capacity (84 kN·m) according to Eurocode 4 [21], except for the specimen CB2M which was tested at a 40% design capacity (48 kN·m). The test arrangement is shown in Figure 3. The composite beams were simply supported and tested in the reversed position where the concrete slab was at the bottom, to simulate the negative moment loading condition in practical engineering. The concentrate loading was applied on the distributive beam by the jack with a capacity of 500 kN. Three Linear Variable Displacement Transducers (LVDT) were placed at the mid-span and two ends, respectively, in order to obtain the deflection during the tests. The pre-load test was first conducted at a 10% design capacity to ensure all the equipment worked normally. All the composite beams were tested under the load control with each load-level at 20 kN to the specified load-level, i.e., 40% or 70% of the design capacity. The load was sustained for approximately 4 minutes before the next loading stage, and the crack development and width were recorded during this period.

After loading, all composite beams with load-induced cracks were moved into the artificial environmental simulation chamber in Zhejiang University. The specimens were exposed to an aggressive chloride environment, with the 5% NaCl solution fog (salt-fog) being generated by sprays that were uniformly located at the top of the chamber. In addition, wet–dry cycles were also simulated as a 4 h long spraying followed by a 44 h drying, at a constant temperature of 45 °C, i.e., a wet–dry cycle lasted two days.

### 2.4. Cracking Maps

The crack propagations and the maximum crack openings were recorded for each pre-gridded concrete slab following the stable development of cracks at each load level. The crack widths were measured using a crack detection machine (model number HC-CK101, made in China) with a measuring range and accuracy of 0.02–2.00 mm and 0.01 mm, respectively. The crack detection machine automatically recognizes the crack width when the sensor is placed and moved on the concrete surface. Several crack-width values for an individual crack under the specific load-step were obtained, and the maximum one was used to make the cracking map in the following Section 2.5.

### 2.5. Assesment of Cross-Section Loss 

The corrosion distribution of steel grids and shear studs was assessed in terms of cross-section loss. When each composite beam finished a pre-set number of wet–dry cycles in the chloride environment, both the reinforcement grids and studs were retrieved. The corrosion products were cleaned according to the procedure in ASTM G1-03 [22].

From a mechanics perspective, the reinforcement is subjected to an axial force, where the deterioration of each cross-section will affect the mechanical performance of the composite beams; however, for the stud shear connectors, the critical section is at the bottom, where the shear fracture can be expected to occur during the ultimate state. Therefore, two measurements were adopted for the reinforcement and for the shear studs.

For the reinforcement, the cross-section loss was assessed by the relative mass loss from the cleaned unit rebars using Equation (1), since it was difficult to measure the diameter of the ribbed bars:

Δ*A* = *A*_0_(*m*_0_ − *m*_1_)/*m*_0_ (1)

where *A*_0_ and *m*_0_ were the nominal cross-section area and the weight per unit length, respectively, measured from the reference non-corroded rebars; and *m*_1_ was the measured residual weight per unit length from the cleaned retrieved reinforcement. The longitudinal reinforcement was cut into 19 sections, so that there were two 50 mm long sections from the ends and seventeen 100 mm ones from the remaining part; whilst the transverse reinforcement was cut into 90 mm per unit lengths.

**Figure 4:**
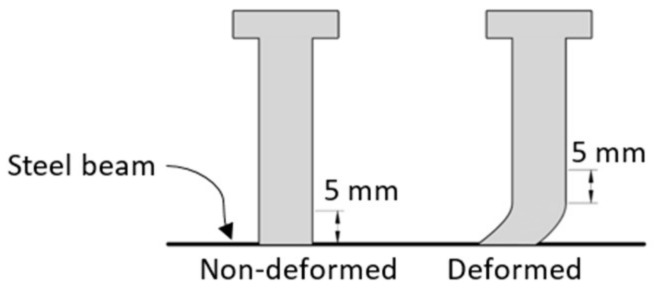
Schematic diagram of measured regions of diameter changes.

For the shear studs, the cross-section loss was assessed by the diameter change from the cleaned unit rebars using Equation (2):

Δ*A* = *A*_0_ − 0.25π*D*_1_^2^ (2)

where *A*_0_ was the cross-section area measured from the reference non-corroded stud shank; and *D*_1_ was the average diameter within the measured region from the cleaned retrieved reinforcement. The measured regions were selected to (1) avoid the shear deformation areas; (2) and manifest the corrosion influence on the moment resisting performance of the composite beam. Therefore, typical measured regions for both deformed and non-deformed scenarios, are shown in Figure 4.

## 3. Experimental Results and Discussion

### 3.1. Crack Maps

**Figure 5:**
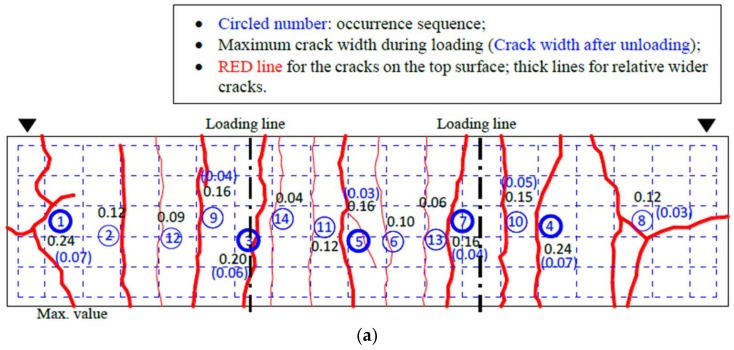

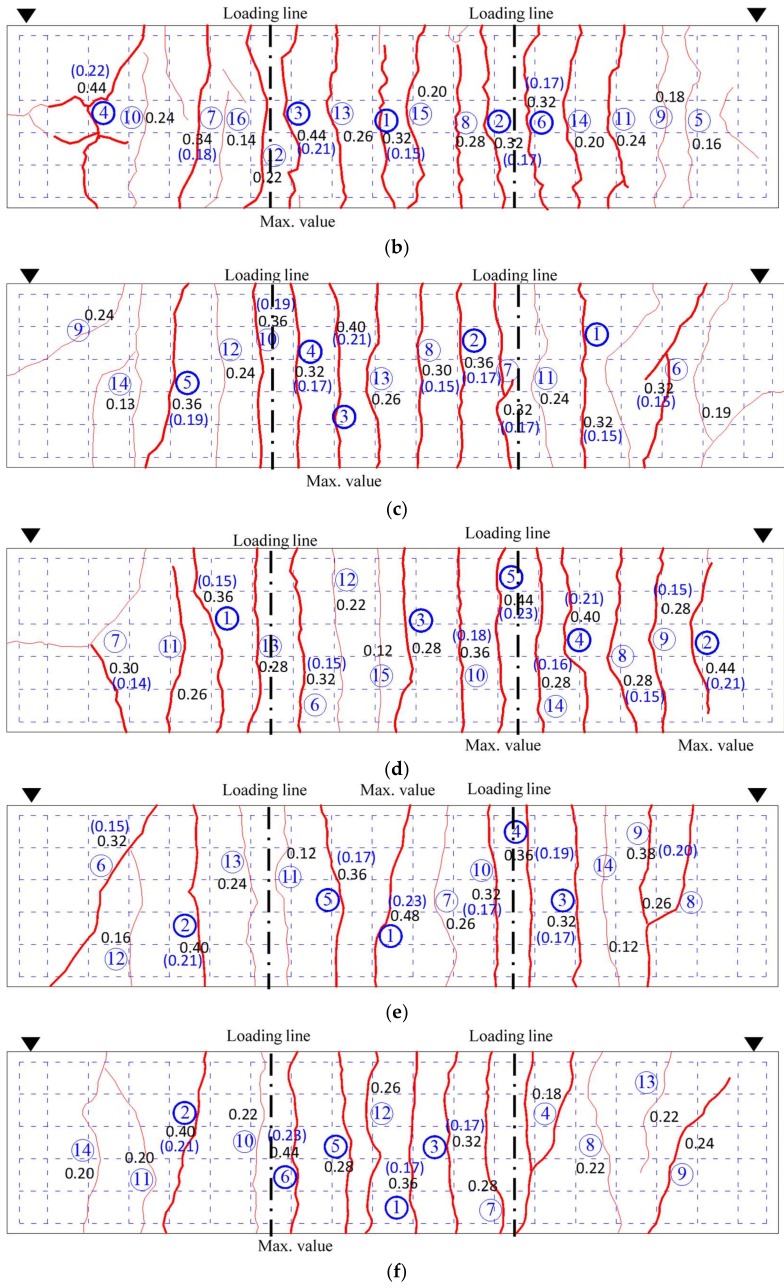

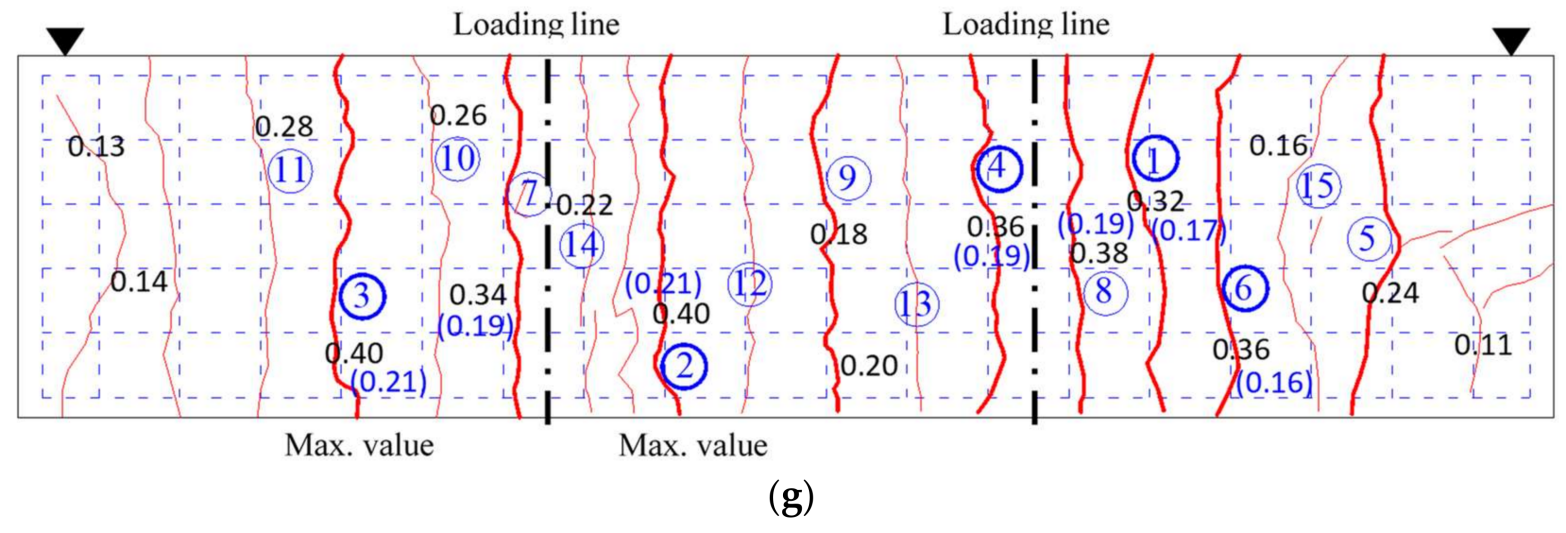
Load-induced crack maps of composite beams: (**a**) CB2M; (**b**) CB4M; (**c**) CB6M; (**d**) CB8M; (**e**) CB10M; (**f**) CB12M; (**g**) CB12MR.

During the pre-load tests, the crack widths and evolutions for the top surface (tensile zone) of the concrete slabs were recorded. It was observed that the first transverse cracks for each composite beam developed within the bending region or the concentrate loading regions at 10% ~ 15% of the design capacity calculated according to EC-4 [21]. As the load increased, a few diagonal cracks developed near two supports in the shear-tension zone. At 60% of the design capacity, the flexure cracks developed at approximately the same spacing of the transverse reinforcement. One can assume that the cracks on the concrete slabs were fully developed after the loading. The crack maps at the maximum load step were recorded as shown in Figure 5, where the cracks were presented in red lines (thick lines for the relatively wider cracks). In the crack maps, the crack propagation is indicated by the circled number in blue which manifests the occurrence sequence of cracks; whilst the maximum openings for each crack are presented via a black number. Moreover, in the parentheses, the maximum widths of typical cracks after unloading (number in blue) were also presented, as shown in Figure 5. It can be found that the maximum crack widths at the top surface (tensile zone) under and after loading were approximately 0.45 mm and 0.25 mm, respectively, at the mid-span of the composite beam. It should be noted that although the seven composite beams were of the same design and constructed using the same materials, the crack width and morphology were different for each of them, which would lead to the differing influences on the corrosion development.

### 3.2. Corossion Patterns

**Figure 6:**
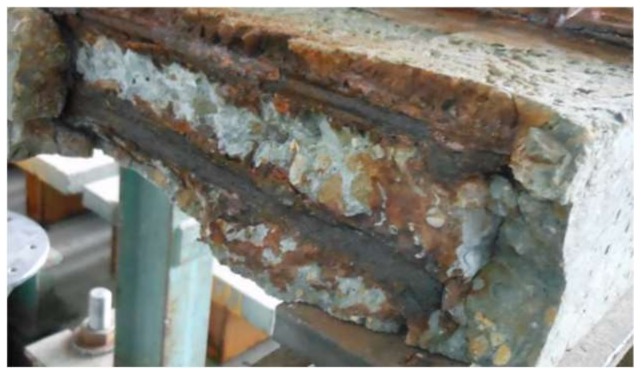
Concrete spalling of specimen CB12M.

**Figure 7:**
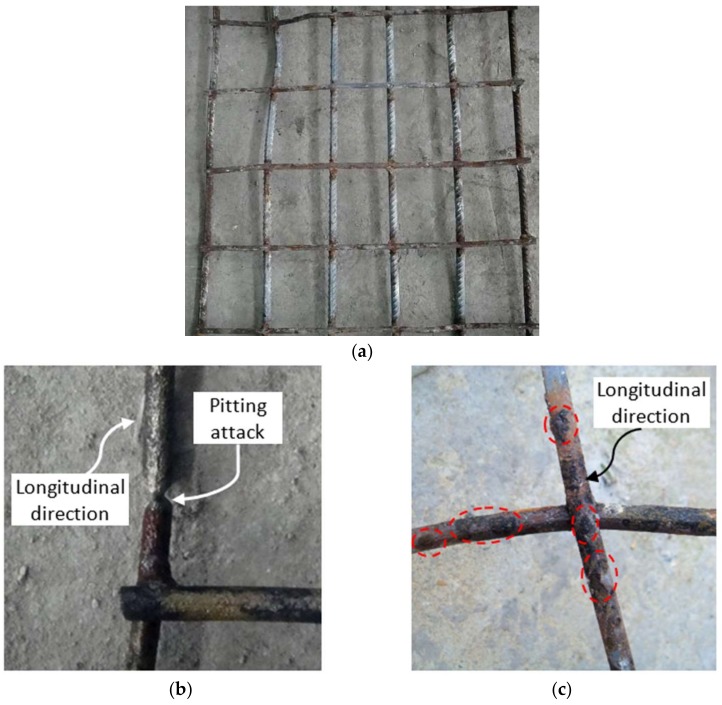
Corrosion pattern for the reinforcement grid in specimens CB6M and CB12M. (**a**) Upper reinforcement grid for CB6M; (**b**) Bottom reinforcement grid for CB6M; (**c**) Bottom reinforcement grid CB12M.

As anticipated, the corrosion increased for both the reinforcement and shear studs with the number of wet–dry cycles. Figure 6 shows the corner concrete spalling from the concrete slab due to the severe corrosion of reinforcement after 12-month’s exposure in the chloride environment combined with wet–dry cycles. However, there is no direct correlation between the location of transverse load-induced cracks and the corrosion development on both the reinforcement and studs of each specimen. Both general and pitting corrosion could be observed, as shown in Figure 7a,b. Moreover, severe pitting attacks combined with general corrosion could be found even in the bottom grids for specimen CB12M, as shown in Figure 7c.

For the transverse reinforcement, the corrosion developed unevenly along the length of the beam and had some relationship with the locations of both wider cracks and concentrate loadings. The transverse reinforcement suffered from the more severe corrosion near the concentrate loading points, such as at the two ends and down 1/3 of the beam-span, and also from cracks with large widths, which can be identified in terms of the values of cross-section loss in the following section. For an individual transverse reinforcement, the corrosion initiated at the cross points is shown in Figure 7a for specimen CB6M.

For the longitudinal reinforcement, the severe corrosion regions were first developed in the points crossed with the transverse ones, as shown in Figure 7a,b for the specimens subjected to 6-month wet–dry cycles, which differed significantly from the findings [23] linked to the reinforced concrete beams. It was found [23] that for the crossing points in the reinforcement cage, the rebars suffered less from the corrosion attack when compared with the vicinity, since in that case the contacted stirrup would be sacrificed as a cathodic protection. However, this did not work in the case of composite beams due to the different connecting methods used in the reinforcement grids. It could be attributed to the spot-welding used in the connection of reinforcements in two directions. During the welding process, the chemical properties, microstructures and connecting surface will be significantly altered [13], making steel more susceptible to a corrosion attack, which will be elaborated on in the following Section 3.3.

**Figure 8:**
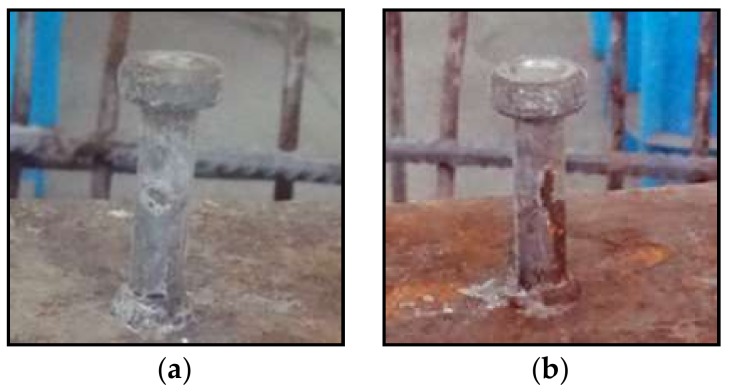

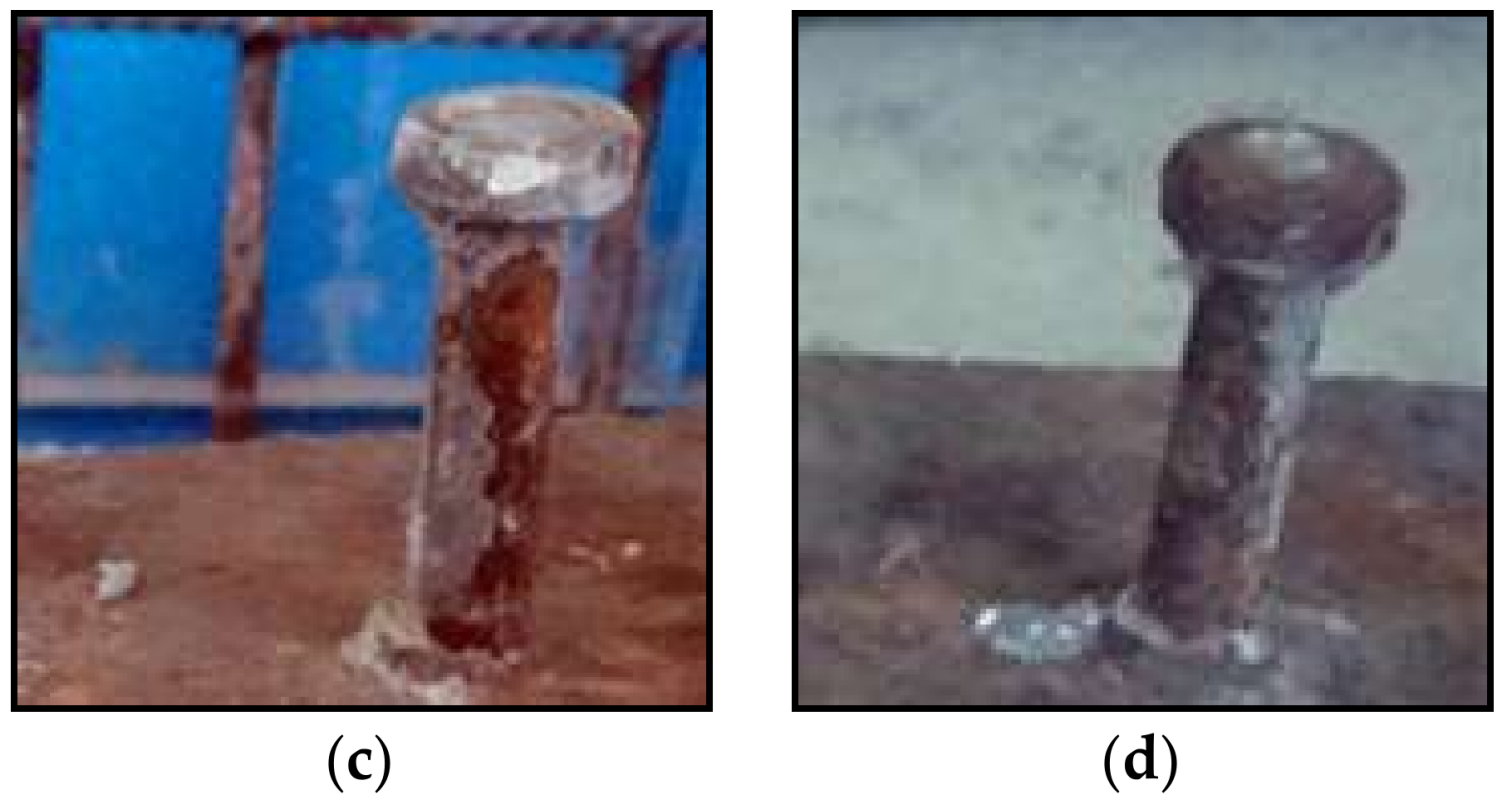
Different corrosion rates of studs in specimen CB6M. (**a**) 7th; (**b**) 2nd; (**c**) 3rd; (**d**) 17th.

Regarding the corrosion development of shear studs, both the distributions along the beam length and the stud shank were quite heterogeneous, as shown in Figure 8. Figure 8 presents the 7th, 2nd, 3rd, and 19th shear studs in the same test specimen of CB6M with corrosion degrees of 0.01%, 5.37%, 5.42% and 8.62%, respectively, and with different patterns, an unevenly distributed corrosion along the height and circumference of the shank. The severe pitting corrosion was also observed in the stud shank. For each individual stud, the corrosion occurred from the bottom shank welded with the steel beam and propagated upwards to the stud head. It could be attributed to the welding connection between the studs and the steel beam, and to the concrete defects or damaged concrete resulting from the complicated surface at the weldment regions, which will be demonstrated in the following Section 3.3.

### 3.3. Cross-Section Loss Distribution

#### 3.3.1. Steel Grids

**Figure 9:**
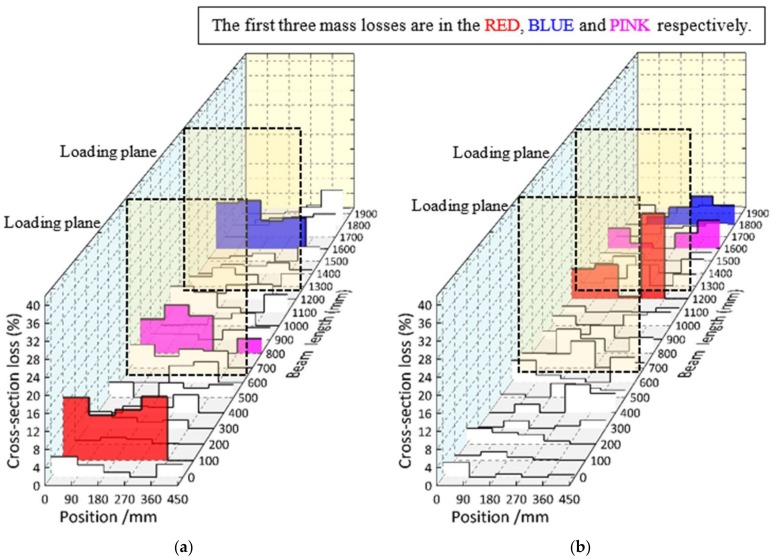

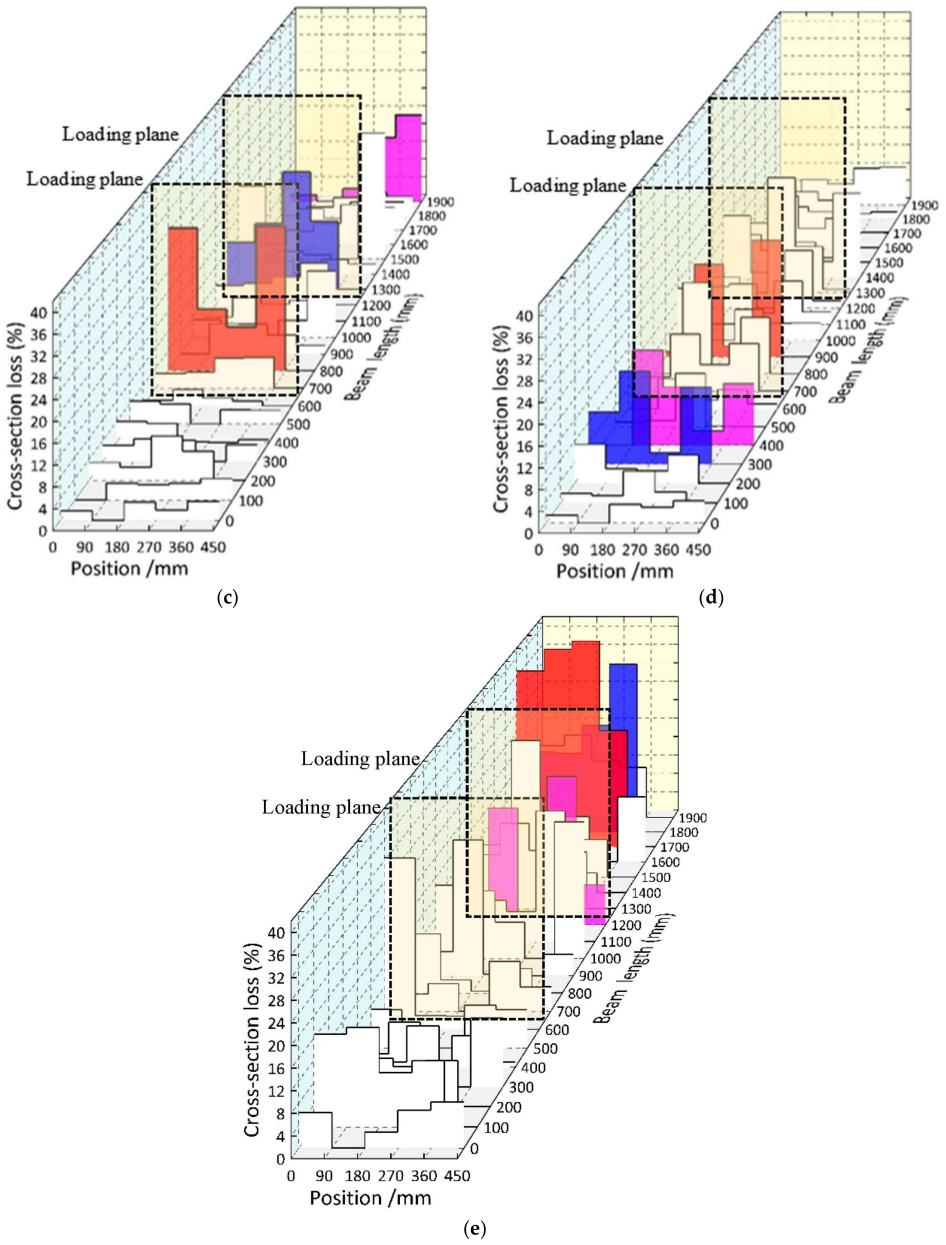
Cross-section loss distributions of the transverse reinforcement of the upper layer. (**a**) CB2M; (**b**) CB4M; (**c**) CB10M; (**d**) CB12M; (**e**) CB12MR.

**Figure 10:**
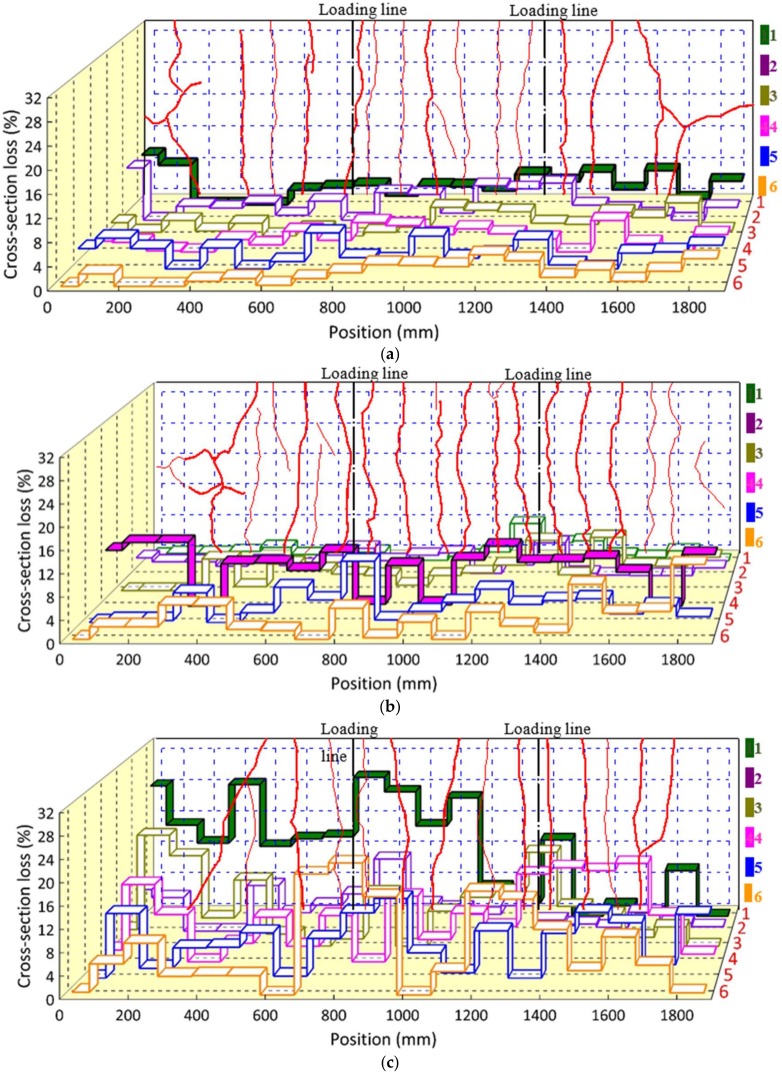

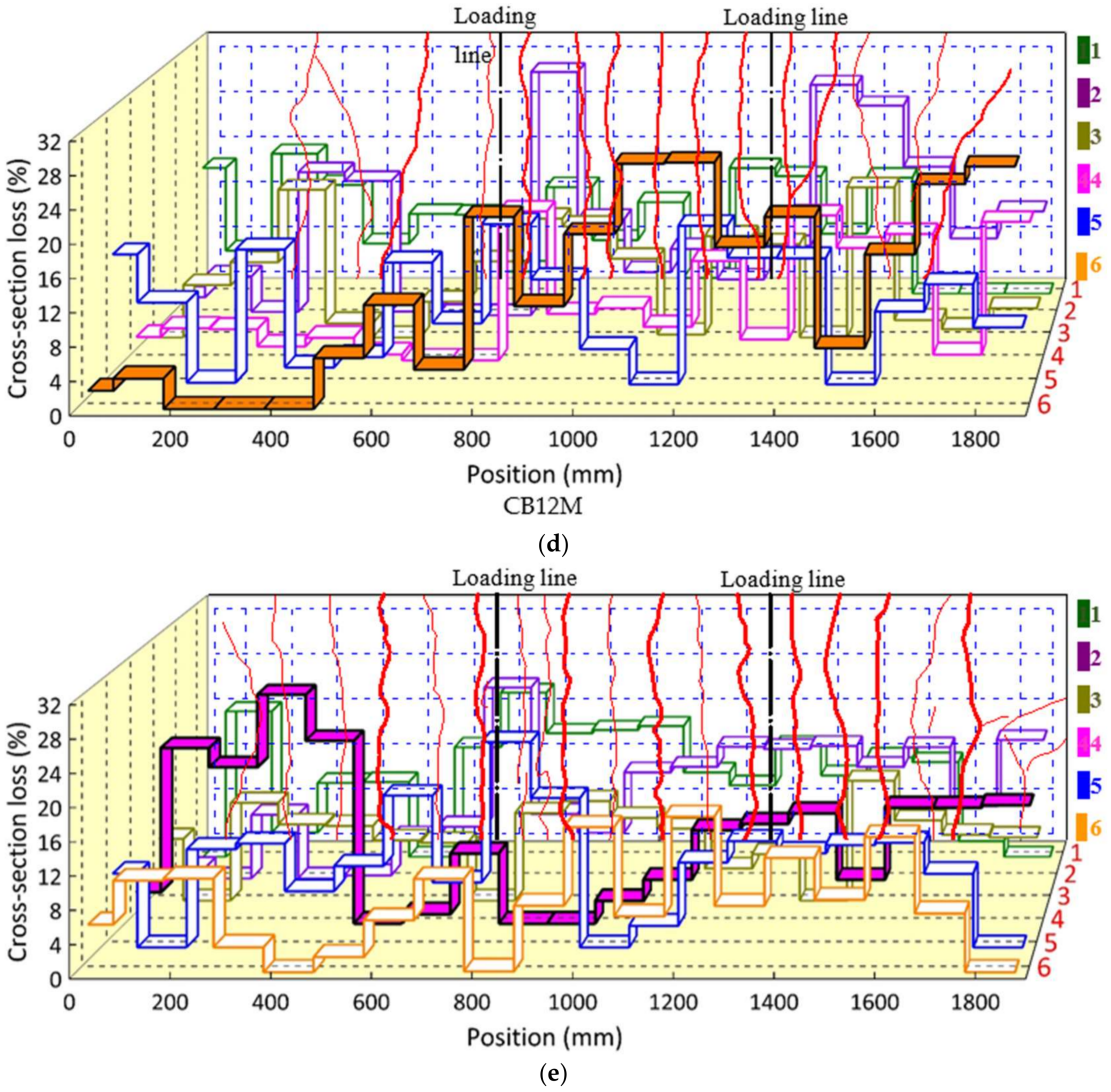
Cross-section loss distributions of the longitudinal reinforcement of the upper layer. (**a**) CB2M; (**b**) CB4M; (**c**) CB10M; (**d**) CB12M; (**e**) CB12MR.

The cross-section loss distributions at different positions of both the transverse and longitudinal reinforcement of the up grids are present in Figure 9 and Figure 10, respectively. The relative mass loss, with respect to the average value, increased with the exposure times in the chloride environment. Discrepancies in the cross-section loss distributions were obtained in specimens CB12M and CB12MR, even though they were of the same design and had the same exposure time.

In Figure 9, the first three mass losses of the transverse reinforcement are indicated in red, blue and pink. The maximum cross-section losses were 14.45%, 22.55%, 29.05%, and 31.95% (the average value of two specimens) for composite specimens subjected to 2, 4, 10, and 12 months, respectively. Meanwhile, the average cross-section losses of the individual transverse reinforcement were 3.04%, 2.22%, 4.77%, and 7.21% for those specimens, respectively. The significantly non-uniformly distributed corrosion for the transverse reinforcement, even after a 12-month exposure, is demonstrated here.

In Figure 10, the longitudinal rebar with the largest average cross-section loss value is highlighted in the solid color bands for each specimen. It was found that the largest cross-section loss of rebar was generally located near the side of the slab where the chloride ion can easily access the rebar from two directions. Moreover, the relationship between the distribution of load-induced cracks and the cross-section loss are presented. There is no direct evidence for the strong correlation between the maximum transverse crack-width and the maximum mass loss. The maximum cross-section losses were 9.45%, 12.93%, 26.20%, and 29.3% (the average value for two specimens) for composite specimens subjected to 2, 4, 10, and 12 months, respectively.

#### 3.3.2. Shear Studs

**Figure 11:**
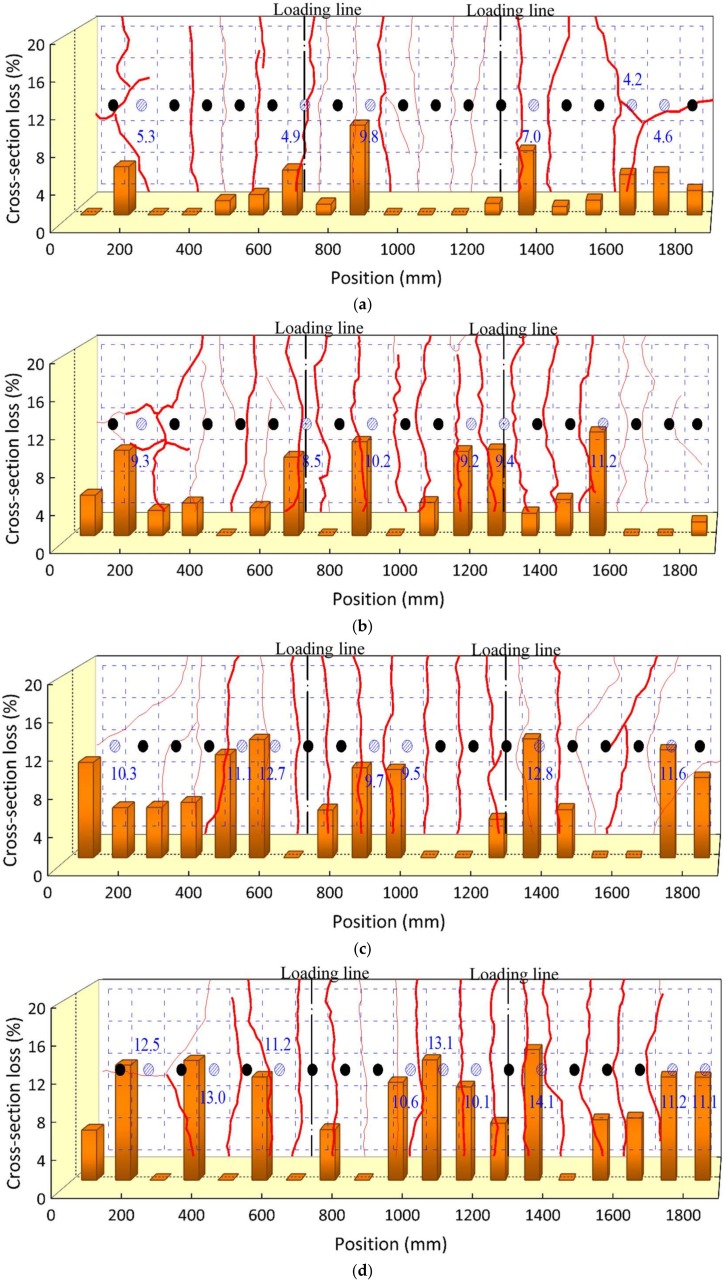

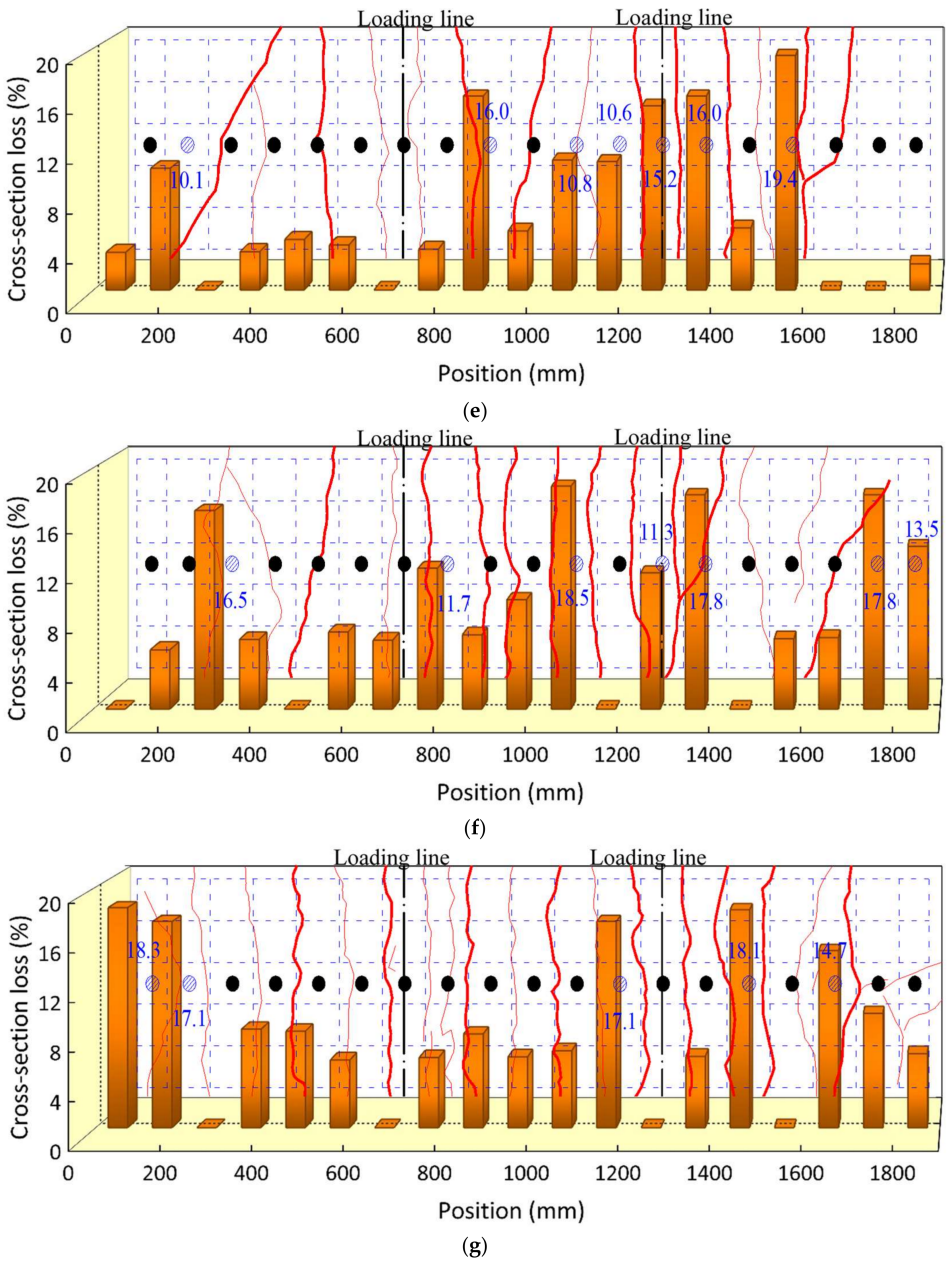
Cross-section loss distributions of the studs. (**a**) CB2M; (**b**) CB4M; (**c**) CB6M; (**d**) CB8M; (**e**) CB10M; (**f**) CB12M; (**g**) CB12MR.

The distributions of the cross-section loss of each shear stud along the length are presented in Figure 11. The cross-section loss with respect to the average value increased with the exposure times. The locations of the studs with a relative large cross-section loss for each specimen are marked in blue, along with the corresponding loss values. These locations seem to have occurred in a random pattern independent from the location of the load-induced cracks. The maximum mass losses were similar for specimens subjected to 10 and 12 months, at an approximate value of 19%. It should be noted that this similarity in the maximum cross-section loss may be due to the measured location as described in the experimental program.

### 3.4. Influenceing Factors on the Corrosion Development on Shear Studs

#### 3.4.1. Load-Induced Cracks

The larger widths of the initial load-induced transverse cracks were not directly responsible for the location of a larger cross-section loss for both the transverse and longitudinal reinforcements and shear studs. Similar observations in RC beams regarding the irrelevance between the locations of the transverse cracks with a large opening and the corrosion rates and propagations of rebars, were also found in the investigation on reinforced concrete beams [1,23,24]. For the longitudinal reinforcement, there was no obvious relationship between the severe mass-loss region and the transverse crack development, while the diagonal cracks in the tension-shear zone normally caused the severe corrosion, especially at the crossed-crack regions. This can be attributed to the fact that the diagonal crack can effectively bring chloride irons and oxygen into the vicinity of the concrete surrounding the longitudinal rebar. Regarding the experimental results, the 3-dimensional crack morphology may have some correlation with the corrosion development since the top surface pattern is a 2-dimensional form, while the transportation of chloride ions and oxygen occurs in a 3-dimentional way. The transportation channel, i.e., the crack propagation underlying the surface crack pattern, can hide the real destination of those detrimental substances, thus leading to a phenomenon of non-correlation between the crack map and the corrosion development. Therefore, a study of the influence of the 3-dimensional crack morphology on the corrosion development should be conducted in the future.

#### 3.4.2. Welding Effects

**Figure 12:**
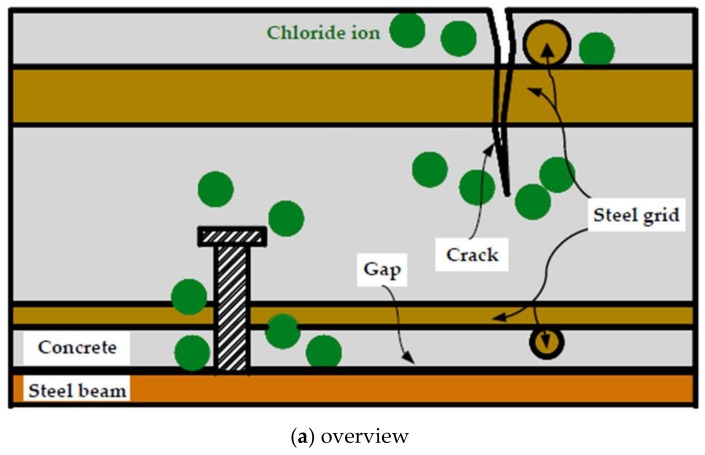

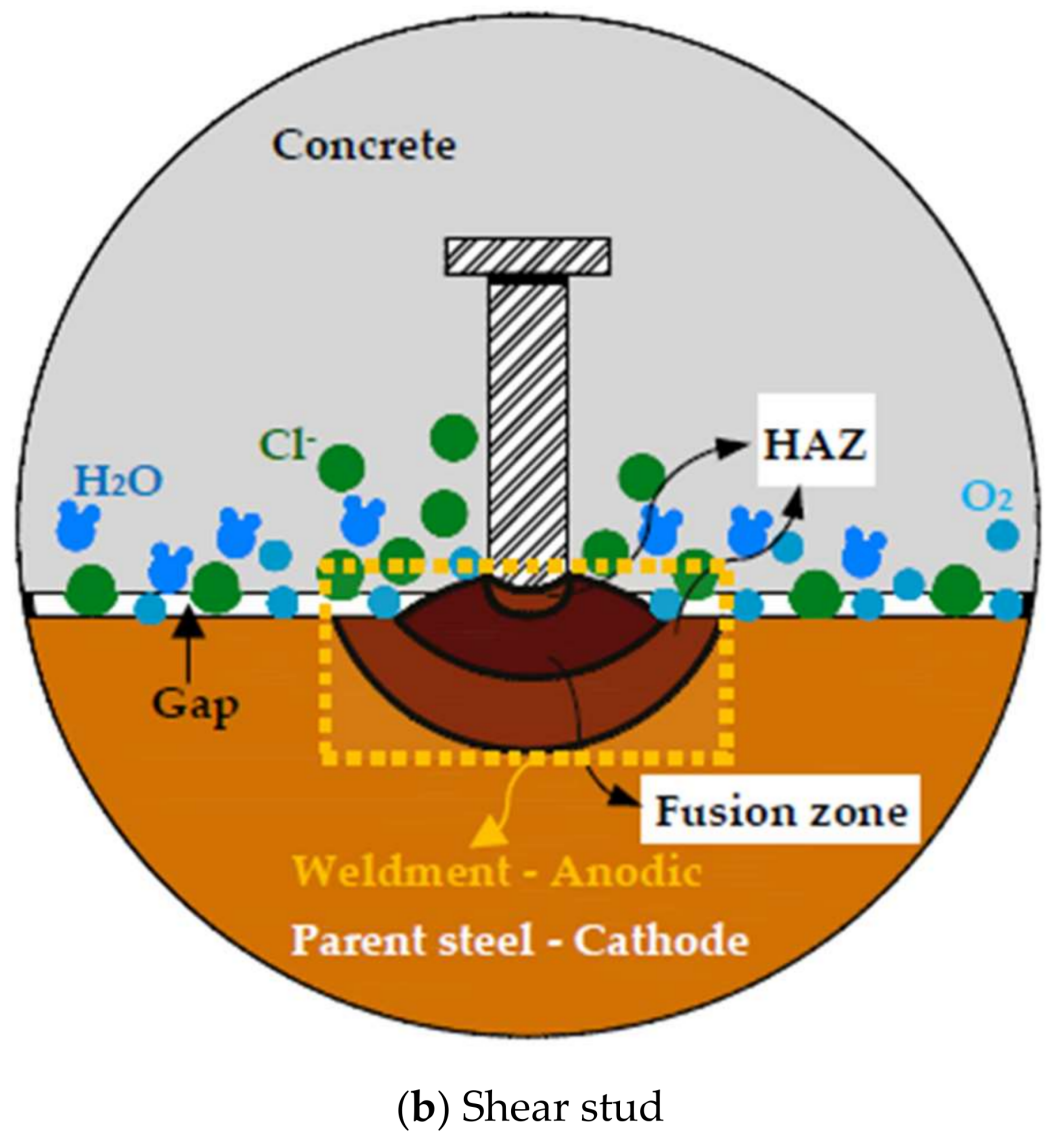
Preferential weldment corrosion in the bottom of the stud. (**a**) overview; (**b**) Shear stud

The preferential corrosion attacks were observed in the steel grids and shear studs shown in Figure 7 and Figure 8 respectively. They can be attributed to the galvanic corrosion. The different electrochemical potentials are found due to variations in the composites and microstructures in the weldments [13]. Moreover, the thermal history during the welding process will bring to each position of the heat-affected zone (HAZ) a unique microstructural feature with its own corrosion susceptibility. The small potential difference between the weldment (as the anodic), including the weld metal and HAZ, and the un-coated parent steel (as the cathode), leads to the formation of the macro cell around these regions, as shown in Figure 12.

The pitting corrosion was also demonstrated, as shown in Figure 7b,c. It can be attributed to the rough surface of the weldment covered by the oxide scale, welding slag and flux residue. These would make the weldments more prone to a localized attack due to the defects developed in the steel–concrete interface, where the electrical conductivity at the crack peak became relatively high when compared to the surrounding intact concrete.

It should be noted that the preferential weldment attack does not refer to the cathode protection for the parent steel. From the perspective of a mechanical performance, it is detrimental for the composite beams to be severely corroded in the connecting region of the shear studs. To avoid the issue of preferential weld metal corrosion attacks, the suitable weld consumables should therefore be selected while taking into consideration both the mechanical properties and corrosion resistances when used in the welded stud shear connectors. Moreover, propriate welding processes and procedure should be adopted [13] to mitigate the preferential weldment corrosion attacks.

## 4. Conclusions

Experimental investigation was performed on the corrosion development of seven cracked steel–concrete composite beams under a chloride environment. Seven composite beams were subjected to a negative moment loading in order to generate the cracks on the concrete slab, and the crack evolutions and maximum widths were obtained. The corrosion pattern and the cross-section loss of the steel grids and shear studs were assessed for the pre-cracked specimens under each specified number of wet–dry cycles. We showed that the corrosion on the grids and studs increased with the exposure number of wet–dry cycles. Both pitting and general corrosion were observed during the exposure period. There is no strong correlation between the severe corrosion regions and the load-induced transverse crack width, for either the reinforcement or the studs. For the transverse reinforcement, the severe cross-section loss generally occurred in the vicinity of the concentrated loadings. Meanwhile the large cross-section loss of the longitudinal reinforcement tended to occur in the tension-shear region, where some diagonal cracks developed. The preferential weldment corrosion caused by galvanic corrosion was evidenced in the steel grids and shear studs at the crossing points and bottom parts, respectively. It can be attributed to the difference of electrochemical potentials between the weldments and the parent steel, leading to the formation of a macro cell. Suitable weld consumables and welding processes should be selected, while taking into consideration both the mechanical properties and corrosion resistances of composite beams subjected to aggressive chloride environments.

## Figures and Tables

**Table 1:** Composite beams subjected to wet–dry cycles.

Specimen	Duration (Month)	Number of Wet–Dry Cycles
CB2M	2	30
CB4M	4	60
CB6M	6	90
CB8M	8	120
CB10M	10	150
CB12M	12	180
CB12MR	12	180

**Table 2:** Mix proportion of concrete.

Water (kg/m^3^)	Cement (kg/m^3^)	Sand (kg/m^3^)	Aggregate (kg/m^3^)
210.0	525.0	524.4	1114.4

**Table 3:** Mechanical properties of steel.

Types	Nominal Diameter (mm)	Measured Area (mm^2^)	Yield Strength (MPa)	Ultimate Strength (MPa)	Elastic Modulus (GPa)	Elongation at Fracture δ_5_ (%)
Stud	10	75.38	462.7	521.2	194.0	26.4
Steel Beam	—	—	333.6	478.7	201.0	38.7
HPB235	6	28.80	298.5	381.2	194.0	30.0
HPB235	8	52.30	427.3	617.0	196.0	29.2
HRB335	10	80.75	447.6	594.2	200.0	33.5
